# Additive interactions of some reduced-risk biocides and two entomopathogenic nematodes suggest implications for integrated control of *Spodoptera litura* (Lepidoptera: Noctuidae)

**DOI:** 10.1038/s41598-020-79725-w

**Published:** 2021-01-14

**Authors:** Rashad Rasool Khan, Muhammad Arshad, Asad Aslam, Muhammad Arshad

**Affiliations:** 1 Department of Entomology, University of Agriculture, Faisalabad, 38000 Pakistan; 2 Plant Protection Research Center, Directorate General of Agriculture and Livestock Research, Ministry of Agriculture, Fisheries Wealth and Water Resources, Muscat, 121 Sultanate of Oman; 3 School of Forestry, Northeast Forestry University, Harbin, 150040 China; 4 Department of Entomology, University of Sargodha, Sargodha, 40100 Pakistan

**Keywords:** Zoology, Entomology

## Abstract

Higher volumes of conventional and novel chemical insecticides are applied by farmers to control resistant strains of armyworm (*Spodoperta litura*) in Pakistan without knowing their risks to the environment and to public health. Ten reduced-risk insecticides were tested for their compatibility with two entomopathogenic nematodes (EPNs); *Heterorhabditis indica* and *Steinernema carpocapsae* against *S. litura*. The insecticide emamectin benzoate was highly toxic (LC_50_ = 2.97 mg/l) against 3^rd^ instar *S. litura* larvae when applied alone whereas, novaluron and methoxyfenozide were the least toxic (LC_50_ = 29.56 mg/l and 21.06 mg/l), respectively. All the insecticides proved harmless against the two EPNs even 96 h after treatment. Indoxacarb, flubendiamide and spinetoram produced the greatest mortalities (72–76%) of *S. litura* larvae after 72 h when applied in mixtures with *H. indica*. Lowest mortalities (44.00 ± 3.74% and 48.00 ± 2.89) were observed for mixtures of *H. indica* with methoxyfenozide and chlorfenapyr, respectively. The positive control treatments with both EPNs (*S. carpocapsae* and *H. indica*) produced > 50% mortality 96 h after treatment. For insecticide mixtures with *S. carpocapsae*, only indoxacarb produced 90% mortality of larvae, whereas, indoxacarb, flubendiamide, emamectin benzoate, and spinetoram produced 90–92% mortality of larvae when applied in mixtures with *H. indica*. Additive interactions (Chi-square < 3.84) of EPN mixtures with reduced volumes of reduced-risk insecticides suggest opportunities to develop more environmentally favorable pest management programs for *S. litura*.

## Introduction

*Spodoptera* spp. (Lepidoptera: Noctuidae), commonly known as armyworms, are polyphagous insect pests, causing serious losses to many cash crops like cotton, maize, tobacco, groundnut, vegetables, legumes and fodder crops in Pakistan^1–3^. Armyworms damage plants by feeding on leaves and neonate larvae feed mainly on epidermal leaf tissue. Leaves with holes are the typical damage symptom of armyworm feeding and at high larval density complete defoliation is possible^4^.

Numerous insecticides belonging to conventional (organophosphates, carbamates, and pyrethroids) and novel classes (insect growth regulators, diamides, spinosyns, and avermectins) are extensively sprayed by farmers to control this pest in Pakistan^5^. Imprudent over-spraying of pesticides may elevate risks to the environment, impair non-target biodiversity and threaten public health^6^. Intensive spraying and repeated use of insecticides from the same chemical group often results in the development of resistance in this pest^4,7,8^. Insecticide resistance in armyworm populations to deltamethrin, cypermethrin, chlorpyrifos, profenofos, spinosad, emamectin benzoate, abamectin, indoxacarb, lufenuron, methoxyfenozide, chlorfluazuron, flufenoxuron, triflumuron, flubendiamide and spinetoram has been reported in Pakistan^2,3,5^.

The development of alternative strategies is badly needed to overcome resistance in this pest. Biopesticides with high selectivity including entomopathogenic nematodes (EPNs), entomopathogenic fungi (EPFs), entomopathogenic bacteria (EPBs), and nuclear polyhedrosis viruses (NPVs) are frequently reported to be an efficient tool in integrated pest management programs^9^. Entomopathogenic nematodes are recognized as important microbial control agents for certain pests of economic significance^10–13^. EPNs belonging to genera *Steinernema* and *Heterorhabditis* are the most studied for their potential to control certain pests like armyworms (*Spodoptera* spp.)^7,11,12,14–16^, diamondback moth (*Plutella xylostella*)^17,18^, tomato leafminer (*Tuta absoluta*)^19–21^, wax moth (*Galleria mellonella*), pink bollworm (*Pectinophora gossypiella*), eggplant fruit borer (*Leucinodes arbonalis*)^13^, oriental beetle (*Anomala orientalis*), Japanese beetle (*Popillia japonica*), native masked chafer (*Cyclocephala borealis*)^22–24^ and white grubs (*Holotrichia parallela* Motschulsky)^24^.

The compatibility of these EPNs with many insecticides has been widely studied against *S. litura*. Reports on toxicity of certain conventional and non-conventional insecticides with novel chemistries indicate that the EPNs (*S. carpocapsae* and *H. indica*) possess significant potential for controlling many insect pests of economic significance in agriculture^12,15,18–21,25^.

Considering the pest status of *S. litura* in Pakistan, and the challenge to overcome the resistance development and pesticide pollution, we investigated the compatibility of some selected insecticides with two EPNs viz., *S. carpocapsae* and *H. indica* as part of an effective pest management strategy. Toxicity of the selected insecticides to *S. litura* larvae were determined alone and in mixtures with *S. carpocapsae* and *H. indica* under laboratory conditions.

## Materials and methods

All insect rearing (*Galleria mellonella* and *Spodoptera litura*), culturing of EPNs (*Heterorhabditis indica* and *Steinernema carpocapsae*) and bioassays were performed in the Insect Diversity and Biosystemics Laboratory of the Department of Entomology, University of Agriculture, Faisalabad, Pakistan.

### Entomopathogenic nematodes (EPNs)

The two entomopathogenic nematode species *S. carpocapsae* and *H. indica* were reared on the late instar larvae of wax worms in the laboratory^12,26^. Entomopathogenic nematodes were collected from dead wax worms using the white trap method, modified for the collection of nematodes^27^. After 8–20 days of infection, infective juveniles emerged from the cadaver when incubated at 20–27 °C. Infective juveniles moved down to water through filter paper and these juveniles were harvested every day until no juveniles were present in the cadaver. All infective juveniles were rinsed from containers and were transferred to a beaker (100 ml). To obtain a clean suspension, the solution was diluted with distilled water by filling the beaker to the top, and nematodes were then collected and stored at 10–15 °C.

### Insects

Wax worms, *G. mellonella*, were collected from infested beehives, and resulting adults were released in plastic jars, measuring 5 cm diameter and 30 cm depth. These jars were provided with cotton swabs soaked with a 10% honey solution for moth feeding and folded card sheets for oviposition. Newly emerged larvae were reared on a wheat-based semi-natural diet, in the laboratory following the mass-rearing technique reported by Khan et al.^28^ and Ashfaq et al.^29^.

Although extensive field surveys were performed to collect *S. litura* larvae, we were only successful in collecting insects from the research fields (Lat. 31.437778°; Long. 73.063611° and Lat. 31.390556°; Long. 73.018056°) of the University of Agriculture, Faisalabad. Upon reaching the laboratory, larvae were reared on washed and dried, fresh leaves of cotton, *Gossypium hirsutum* L., and kept in an insect rearing chamber at 26 ± 2 ºC, 75 ± 10% RH, and 12:12 LD. Field collected adults and those emerging from pupae in the laboratory were kept in well-ventilated cages (30 × 30 × 30 cm) and provided a cotton swab dipped in a sucrose-solution (100 g/l), also containing vitamin solution (20 ml/l) and methyl 4-hydroxybenzoate (2 g/l) for adult feeding ^3,8,12^. The homogenous lots of insects were used for bioassays with EPNs and insecticides.

### Insecticide formulations

Ten insecticides with novel modes of action were selected for evaluation along with the selected EPN species against *S. litura*. All of the selected insecticides are registered for use against *Spodoptera* spp. in Pakistan^30^. Further details about the recommended dose rates, active ingredients, mode of action, target pests, and suppliers/distributors in Pakistan are provided in Table 1. The test concentrations of each formulated insecticide were prepared in distilled water and were used in all bioassay tests^1,3,8,31^.

**Table 1:** List of insecticides selected for use against *S. litura* in Pakistan.

Insecticides	Active ingredient	Mode of action	Supplier	Target pests	Field recommended dose against *S. litura*
Chlorantraniliprole	Coragen 20SC	Ryanodine receptor modulator	FMC, Pakistan	*Earias* spp., *Helicoverpa* spp., *Spodoptera* spp., *Chilo* spp., *Plutella* sp.	50 ml/Acre (100 mg/l)
Chlorfenapyr	Pirate 36SC	Uncoupler of oxidative phosphorylation via disruption of the proton gradient	Swat Agro Chemicals	*Spodoptera* spp., *Phyllocnistis citrella*, *Plutella* sp.	100 ml/Acre (360 mg/l)
Emamectin benzoate	Proclaim 019EC	Glutamate-gated chloride channel (Glucl) allosteric modulator	Syngenta, Pakistan	*Earias* spp., *Helicoverpa* spp., *Spodoptera* spp., *Plutella* sp.	200 ml/Acre (38.4 mg/l)
Flubendiamide	Belt 48SC	Ryanodine receptor modulator	Bayer Pakistan, Crop Science Division	*Helicoverpa* spp., *Spodoptera* spp., *Plutella* sp.	20 ml/Acre (120 mg/l)
Indoxacarb	Steward 150EC	Voltage-dependent sodium channel blocker	FMC, Pakistan	*Earias* spp., *Helicoverpa* spp., *Spodoptera* spp., *Plutella* sp.	125 ml/Acre (187.5 mg/l)
Lufenuron	Match 050EC	Inhibitor of chitin biosynthesis	Syngenta, Pakistan	*Earias* spp., *Helicoverpa* spp., *Spodoptera* spp., *Phyllocnistis citrella*, *Plutella* sp.	200 ml/Acre (100 mg/l)
Methoxyfenozide	Runner 240SC	Ecdysone receptor agonist	Arysta Life Science, Pakistan	*Helicoverpa* spp., *Spodoptera* spp.	200 ml/Acre (480 mg/l)
Novaluron	Corvus 10EC	Inhibitor of chitin synthesis	FMC, Pakistan	*Helicoverpa* spp., *Spodoptera* spp.	300 ml/Acre (300 mg/l)
Spinetoram	Radiant 120SC	Nicotinic acetylcholine receptor (Nachr) allosteric modulator	Arysta Life Science, Pakistan	*Earias* spp., *Helicoverpa* spp., *Spodoptera* spp., *Pectinophora gossypiella*	100 ml/Acre (120 mg/l)
Spinosad	Tracer 24SC	Nicotinic acetylcholine receptor (Nachr) allosteric modulator	Arysta Life Science, Pakistan	*Earias* spp., *Helicoverpa* spp., *Spodoptera* spp., *Phyllocnistis citrella, Diaphorina citri*, *Chilo* spp., *Plutella* sp.	80 ml/Acre (288 mg/l)

### Toxicity of insecticides to *S. litura*

Preliminary bioassays were performed to estimate the range of insecticides’ lethal concentrations killing 5–95% of 3rd instar larvae of *S. litura*. Groups of 10 larvae were used for each insecticide treatment, with insecticide solutions applied using a leaf dip method^1,3,8^. A single leaf disc (5 cm diameter) dipped in each insecticide concentration for 10 s and dried on a paper towel, was placed on moist filter papers to avoid desiccation in Petri plates. Each insecticide concentration was repeated five times and leaves were treated separately at different time with new insecticide solution of same concentration prepared on each occasion. The leaves dipped in distilled water alone served as the untreated check in all replicates. All treated and untreated larvae were kept in an insect growth chamber at 26 ± 2 ºC, 75 ± 10% RH, and 12:12 LD. Mortality was determined 96 h after treatment applications. Larvae were considered dead when no movement of appendages was seen upon touching with a dissecting needle.

### Toxicity of insecticides to EPNs

Toxicity of the field recommended dose rates of the selected insecticides was studied against the two species of EPNs to estimate their compatibility with the test chemicals. A series of conventional mortality bioassay tests were performed to determine the toxic effects of the ten insecticides against EPNs under laboratory conditions of 26 ± 2 ºC, 75 ± 10% RH, and 12:12 LD. In a sterilized glass tube, 1 ml insecticide test solution was added along with 10 µl Ringer’s solution containing 100 entomopathogenic infective juveniles (IJs) of the selected species. The treatment solutions were thoroughly mixed by gently tapping the tubes. All treatments were repeated five times and a distilled water treatment was applied as a control. The replications were performed with new insecticide solutions of same concentrations but prepared at a different time under same set of conditions. Non-responding EPNs upon touching with a probe or dissecting needle were considered dead from the treatment^7,12,21,25,26^. Mortality of the IJs was determined after 48, 72, and 96 h. The insecticides resulting in less than 25% mortality were denoted as harmless or least toxic^32^.

### Toxicity of insecticides and EPN mixtures to *S. litura*

The toxicity of ten selected insecticides and the EPN mixtures was evaluated in *S. litura* using previously reported methodology^7,12,26^. Plastic containers (150 ml) were provided with wet sand (10%, w/w) and five grams of insect diet. To the sand and diet in each container was further applied a solution containing the median lethal concentration (LC_50_) of each insecticide (Table 2) and 500 IJs (in 50 μl Ringer’s solution) of each EPN sp., separately. A treatment containing the 500 IJs of each EPN sp., alone served as a negative control for Chi-square calculations. Ten larvae were maintained in each treated container, with five replicates maintained under laboratory conditions at 26 ± 2 ºC, 75 ± 10% RH, and 12:12 LD. Mortality was assessed after 72 and 96 h of larval release into the containers.

**Table 2:** Toxicity of selected insecticides against *S. litura* after 96 h.

Insecticides	n	LC_50_ (95% CI)	Slope (SE)	χ^2^	df	P
Chlorantraniliprole	25	5.50(3.48–7.53)	2.11(0.19)	5.22	3	0.30
Chlorfenapyr	25	17.16(9.41–25.01)	1.74(0.17)	5.69	3	0.32
Emamectin benzoate	25	2.97(2.43–3.51)	1.84(0.17)	2.80	3	0.29
Flubendiamide	25	7.41(4.66–10.29)	1.83(0.17)	4.75	3	0.29
Indoxacarb	25	10.92(7.17–14.69)	2.12(0.19)	4.65	3	0.30
Lufenuron	25	7.85(6.55–9.22)	1.78(0.16)	0.58	3	0.24
Methoxyfenozide	25	21.06(17.02–25.07)	1.84(0.18)	2.91	3	0.34
Novaluron	25	29.56(13.12–42.85)	1.86(0.17)	11.99	3	0.24
Spinetoram	25	9.08(7.60–10.64)	1.83(0.16)	0.80	3	0.24
Spinosad	25	14.78(6.56–24.77)	1.86(0.17)	11.99	3	0.24

*n* number of observations used for Probit analysis, *LC*_*50*_ median lethal concentration (mg/l), *95% CI* 95% confidence interval.

### Statistical analysis

A completely randomized design (CRD) was used for bioassays with insecticides and EPNs. In the case of bioassays with insecticides and *S. litura*, mortality scores were corrected using Abbott’s formula^33^. The corrected mortality values were further subjected to Probit analysis and the median lethal concentrations (LC_50_) were calculated using Polo Plus software Version 1.0 (LeOra Software LLC). The lethal concentrations of various insecticide treatments were reported with significant differences when 95% confidence intervals (CIs) were non-overlapping^34^.

Data for the toxicity of the test chemical insecticides against the selected EPNs were analyzed statistically through Minitab 18 Software and the means were compared for significance using Tukey's HSD test (P < 0.05).

The mean mortality scores obtained in the combined treatments of insecticides and EPNs were also subjected to ANOVA and means were compared for significance as stated above. However, comparisons of the expected versus observed mortality of *S. litura* was performed through a binomial test and the interaction between insecticides and EPN mixtures was described as additive, antagonistic or synergistic ^7,12,20^. Expected larval mortality in each treatment was estimated as:

$${P}_{E}={P}_{^\circ }+\left(1-{P}_{^\circ }\right)\left({P}_{1}\right)+(1-{P}_{^\circ })(1-{P}_{1})({P}_{2})$$

where, P_E_ symbolizes mortality (expected) in mixture treatments of EPNs and insecticides, P_O_ denotes mortality of *S. litura* in the control treatment, P_1_ symbolises the mortality of *S. litura* due to insecticide alone and P_2_ represents the mortality (observed) in the treatment with EPNs alone.

A chi-square (χ^2^) value was calculated by using the formula

$${\upchi }^{2}=\left\{\frac{{\left({L}_{^\circ }-{L}_{e}\right)}^{2}}{{L}_{e}}\right\}+\left\{\frac{{\left({D}_{^\circ }-{D}_{e}\right)}^{2}}{{D}_{e}}\right\}$$

where L_o_ displays the observed numbers of live larvae in the treatment, L_e_ denotes the expected number of live larvae in the treatment, D_o_ is the number of observed dead larvae and D_e_ is the number of expected dead larvae in the treatment.

We tested the hypothesis of treatment interactions by using a 3.84 value of Chi-square (df = n − 1; and P = 0.05). Synergy was assigned to the insecticide and EPN treatments when observed mortality (P_o_) was higher than the expected mortality (P_e_) and the χ^2^ was higher than 3.84. The interaction was considered additive when the χ^2^ value < 3.84 and antagonism was defined as treatment mixtures where observed mortality (P_o_) was less than expected mortality (P_e_) and the χ^2^ was higher than 3.84^7,12,20^.

### Ethical statement

As no human or mammalian subjects were involved in this research, hence, no ethics approvals were required for this study. However, we adapted all the standard bioassay procedures while conducting the experiments.

## Results

### Toxicity of insecticides to *S. litura*

The selected insecticides were tested for their toxicity against the *S. litura* larvae to obtain their relevant median lethal concentrations (LC_50_). These median lethal values were further used in treatment mixtures with the EPN spp. The results of Probit analyses are displayed in Table 2. The insecticide emamectin benzoate was found to be highly toxic (LC_50_ = 2.97 mg/l) against 3rd instar *S. litura* larvae. The highest median lethal concentration (LC_50_ = 29.56 mg/l) was determined for the insecticide novaluron which was followed by methoxyfenozide (LC_50_ = 21.06 mg/l), whereas the toxicity scores of chlorfenapyr (LC_50_ = 17.16 mg/l), spinosad (LC_50_ = 14.78 mg/l) and indoxacarb (LC_50_ = 10.92 mg/l) did not differ significantly from each other because of overlapping confidence intervals. The insecticides chlorantraniliprole, flubendiamide and lufenuron also proved very toxic against 3rd instar *S. litura* larvae and exhibited lower LC_50_ values (5.50, 7.41 and 7.85 mg/l, respectively).

### Toxicity of insecticides to EPNs

A series of conventional mortality bioassay tests were performed to determine the toxic effects of the field recommended dose rates of ten insecticides against EPNs and the results are displayed in Table 3. We found significant variation among all the tested insecticides against both EPN species (*S. carpocapsae* and *H. indica*) after different exposure times (P < 0.05). All the tested insecticides proved to be harmless against the two tested EPN species (*S. carpocapsae* and *H. indica*) based on less than 20% mortality, even after 96 h of treatment exposure, and less than 10% mortality in both EPN spp., after 72 h. The highest mortality (16.27 ± 0.9) of *H. indica* was recorded after 96 h with chlorfenapyr, followed by spinosad (14.89 ± 1.1), flubendiamide (13.37 ± 1.0), spinetoram (13.22 ± 1.0) and indoxacarb (13.21 ± 1.0). Almost the same insecticides viz., chlorfenapyr, spinosad, indoxacarb, spinetoram and flubendiamide proved to be harmless against *S. carpocapsae*, causing mortality ranging from 11.00 to 14.33% after 96 h of treatment.

**Table 3:** Toxicity of selected insecticides against entomopathogenic nematodes.

Insecticides	Dose (mg/l)^a^	EPNs Mortality (%) after					
		48 h		72 h		96 h	
		Sc	Hi	Sc	Hi	Sc	Hi
Chlorantraniliprole	100	0.33 ± 0.14bc *	1.23 ± 0.41bc *	4.33 ± 0.32e *	5.76 ± 0.62e *	9.00 ± 0.91c *	10.89 ± 0.99c *
Chlorfenapyr	360	3.00 ± 0.71a	3.91 ± 0.89a	8.00 ± 0.79ab	9.41 ± 0.81ab	14.33 ± 1.02a	16.27 ± 0.99a
Emamectin benzoate	38.4	2.00 ± 0.46abc	2.90 ± 0.59abc	7.00 ± 0.67abc	8.39 ± 0.81abc	10.33 ± 0.92bc	12.22 ± 0.98bc
Flubendiamide	120	2.67 ± 0.51a	3.71 ± 0.82a	6.67 ± 0.71bcd	8.10 ± 0.87bcd	11.00 ± 1.00abc	13.37 ± 1.03abc
Indoxacarb	187.5	2.67 ± 0.57a	3.57 ± 0.71a	8.33 ± 0.51a	9.76 ± 0.91a	11.33 ± 0.92abc	13.21 ± 1.02abc
Lufenuron	100	2.67 ± 0.49a	3.53 ± 0.69a	7.67 ± 0.62ab	9.08 ± 0.69ab	9.67 ± 0.57bc	11.57 ± 0.87bc
Methoxyfenozide	480	1.33 ± 0.57abc	2.25 ± 0.81abc	4.67 ± 0.39de	6.10 ± 0.89de	9.33 ± 0.71c	11.27 ± 1.01bc
Novaluron	300	2.33 ± 0.42ab	3.27 ± 0.62ab	6.00 ± 0.39cde	7.39 ± 0.79cde	10.67 ± 0.98bc	12.56 ± 1.11bc
Spinetoram	120	2.00 ± 0.49abc	2.97 ± 0.61abc	5.35 ± 0.47cde	6.76 ± 0.71cde	11.33 ± 1.01abc	13.22 ± 1.09abc
Spinosad	288	2.33 ± 0.58ab	3.23 ± 0.67ab	8.00 ± 0.47ab	9.43 ± 0.89ab	13.00 ± 1.00ab	14.89 ± 1.10ab
Control		0.00 ± 0.00	0.00 ± 0.00	0.00 ± 0.00	0.00 ± 0.00	0.00 ± 0.00	0.00 ± 0.00
Statistic summary		F = 05.07, P < 0.001 DF = 10, R^2^ = 69.75%	F = 07.91, P < 0.001 DF = 10, R^2^ = 73.71%	F = 32.27, P < 0.001 DF = 10, R^2^ = 93.62%	F = 27.32, P < 0.001 DF = 10, R^2^ = 91.73%	F = 27.16, P < 0.001 DF = 10, R^2^ = 92.51%	F = 31.91, P < 0.001 DF = 10, R^2^ = 89.71%

*Sc* *S. carpocapsae*, *Hi* *H. indica.*

*Means followed by the same letter within a column do not differ significantly at 5% probability.

^a^Field recommended dose rates against *S. litura* and to each insecticide treatment 100 IJs of selected EPN species were added.

### Toxicity of insecticides and EPN mixtures to *S. litura*

Toxicity of selected insecticides and EPN mixtures were recorded after 72 and 96 h of treatment application. In our study, insecticides proved to be an important factor in *S. litura* mortality, along with the EPNs. The results revealed significant variations in the toxicity levels of all the treatments and exhibited considerable differences in the mortality of *S. litura* larvae in all insecticide mixtures with *S. carpocapsae* (F = 86.15, P < 0.001, df = 11, R-sq. = 95.21%) and *H. indica* (F = 73.67, P < 0.001, df = 11, R-sq = 91.79%) after 72 h of treatment application (Table 4).

**Table 4:** Toxicity of selected insecticides and nematode mixtures against *S. litura* after 72 h of treatment applications.

Insecticides and EPN mixtures ^a^	*S. litura* mortality (%) and interactive response between insecticides and EPNs mixtures					
	Sc			Hi		
	Mortality	χ^2^	Response	Mortality	χ^2^	Response
EPNs (alone)	38.00 ± 1.99d *	–	–	44.00 ± 2.89d *	–	–
Chlorantraniliprole	64.00 ± 3.74ab	1.65	Additive	66.00 ± 2.89ab	0.03	Additive
Chlorfenapyr	44.00 ± 3.74 cd	0.90	–	48.00 ± 2.89 cd	0.13	–
Emamectin benzoate	66.00 ± 2.45ab	1.73	Additive	66.00 ± 3.99ab	0.05	Additive
Flubendiamide	66.00 ± 2.45ab	1.73	Additive	74.00 ± 2.89a	0.05	Additive
Indoxacarb	74.00 ± 1.99a	2.03	Additive	76.00 ± 2.90a	0.12	Additive
Lufenuron	62.00 ± 2.45ab	1.58	Additive	64.00 ± 2.45ab	0.02	Additive
Methoxyfenozide	38.00 ± 3.99d	0.68	–	44.00 ± 3.74d	0.18	–
Novaluron	56.00 ± 1.99bc	1.35	Additive	58.00 ± 2.89bc	0.03	Additive
Spinetoram	68.00 ± 2.45ab	1.8	Additive	72.00 ± 3.74a	0.07	Additive
Spinosad	56.00 ± 2.45bc	1.35	Additive	62.00 ± 2.90ab	0.03	Additive
Control	0.00 ± 0.00e			0.00 ± 0.00e		
Statistic summary	F = 86.15, P < 0.001 DF = 11, R^2^ = 95.21%			F = 73.67, P < 0.001 DF = 11, R^2^ = 91.79%		

*Sc* *S. carpocapsae*, *Hi* *H. indica.*

*Means followed by the same letter within a column do not differ significantly at 5% probability.

^a^Median lethal concentrations (LC_50_) of insecticides and 500 IJs of each EPN specie were applied in each treatment mixture.

No synergistic, nor antagonistic interactions, were observed between the insecticides and EPNs (*S. carpocapsae* and *H. indica*). The highest mortality (74.00 ± 1.99%) was observed with an additive interaction (Chi-sq. = 2.03) when *S. carpocapsae* was applied along with indoxacarb. The insecticides indoxacarb, flubendiamide, and spinetoram produced the greatest mortalities (72–76%) of *S. litura* larvae, when applied in mixtures with *H. indica* after 72 h. The interaction between these insecticides and the EPN sp., (*H. indica*) was found to be additive (Χ^2^ < 3.84). Lowest mortality (44.00 ± 3.74% and 48.00 ± 2.89) was observed in mixture of *H. indica* with methoxyfenozide and chlorfenapyr, respectively. Similarly, the same insecticides exhibited the lowest mortalities (38.00 ± 3.99 and 44.00 ± 3.74%) when applied in mixed form with *S. carpocapsae*. No interaction was determined for either EPN (*S. carpocapsae* and *H. indica*) and methoxyfenozide mixtures, because mortality in the positive control (EPNs only) and the mixture were the same. However, an additive interaction was found in all other insecticide mixtures with *S. carpocapsae* and *H. indica* because of a smaller chi-square value than 3.84 (Table 4). Mixtures of emamectin benzoate, chlorantraniliprole, lufenuron and spinosad with *H. indica* also proved effective against *S. litura* larvae and exhibited an additive interaction between the EPN and insecticides (62–66% mortality and Chi-sq < 3.84).

The results obtained after 96 h also revealed significant variations in toxicity levels of the treatments and exhibited considerable differences in the mortality of *S. litura* larvae in all insecticide mixtures with *S. carpocapsae* (F = 102.76, P < 0.001, df = 11, R-sq = 95.95%) and *H. indica* (F = 93.43, P < 0.001, df = 11, R-sq = 93.91%) after 72 h (Table 5).

**Table 5:** Toxicity of selected insecticides and nematode mixtures against *S. litura* after 96 h of treatment application.

Insecticides and EPNs mixtures^a^	*S. litura* mortality (%) and interactive response between insecticides and EPN mixtures					
	Sc			Hi		
	Mortality	χ^2^	Response	Mortality	χ^2^	Response
EPNs (alone)	51.00 ± 1.91d *	–	–	62.00 ± 2.58 cd *	–	–
Chlorantraniliprole	84.00 ± 2.45ab	1.65	Additive	86.00 ± 2.58ab	0.12	Additive
Chlorfenapyr	64.00 ± 2.45c	0.90	Additive	68.00 ± 2.58 cd	1.32	Additive
Emamectin benzoate	86.00 ± 3.99ab	1.73	Additive	90.00 ± 3.97a	0.12	Additive
Flubendiamide	88.00 ± 1.99ab	1.73	Additive	92.00 ± 1.99a	0.12	Additive
Indoxacarb	90.00 ± 3.16a	2.03	Additive	92.00 ± 3.16a	0.24	Additive
Lufenuron	84.00 ± 2.45ab	1.58	Additive	86.00 ± 2.58ab	0.12	Additive
Methoxyfenozide	64.00 ± 2.45c	0.68	Additive	66.00 ± 2.90 cd	1.91	Additive
Novaluron	76.00 ± 2.45bc	1.35	Additive	78.00 ± 3.16bc	0.60	Additive
Spinetoram	86.00 ± 3.99ab	1.8	Additive	90.00 ± 3.92a	0.96	Additive
Spinosad	76.00 ± 2.45bc	1.35	Additive	80.00 ± 2.97bc	0.59	Additive
Control	0.00 ± 0.00e			0.00 ± 0.00e		
Statistic Summary	F = 102.76, P = 0.00 DF = 11, R^2^ = 95.95%			F = 93.43, P = 0.00 DF = 11, R^2^ = 93.91%		

*Sc* *S. carpocapsae*, *Hi* *H. indica.*

*Means followed by the same letter within a column do not differ significantly at 5% probability.

^a^Median lethal concentrations (LC_50_) of insecticides and 500 IJs of each EPN specie were applied in each treatment mixture.

All the insecticide and EPN mixtures exhibited additive interactions indicating that both insecticides and EPNs contributed significantly to *S. litura* larval mortality (Table 5). However, we did not observe any synergistic or antagonistic interactions between insecticides and EPNs even after 96 h of treatment. The positive controls with both EPN spp., produced more than 50% mortality after 96 h (*S. carpocapsae* = 51.00 ± 1.91% and *H. indica* = 62.00 ± 2.58%). In the case of insecticide mixtures with *S. carpocapsae*, indoxacarb produced 90% mortality of *S. litura* larvae, whereas, indoxacarb, flubendiamide, emamectin benzoate, and spinetoram produced 90—92% mortality when applied in mixtures with *H. indica*. The two insecticides viz., methoxyfenozide and chlorfenapyr produced minimum mortalities (64–68%) with EPN mixtures even after 96 h.

## Discussion

Insecticides are most widely used by farmers to control several key pests of agricultural significance. Many insect pests including armyworms, *Spodoptera* spp., have been reported to develop resistance against the insecticides used in pest management programs^1,2,8^. Combining certain efficient techniques like biological control agents, especially EPNs, and insecticides can help address the challenges in management of certain pest insects in agriculture^7,12,13,21,25,35,36^.

As mentioned earlier, *S. litura* has developed resistance to many of the chemical insecticides used for its management in many countries. In the present study, emamectin benzoate was found to be highly toxic to 3rd instar *S. litura* larvae, whereas, novaluron and methoxyfenozide were the least toxic amongst the tested insecticides. We could not find any report of resistance development in *Spodoptera* spp. against novaluron; however, it has been reported to methoxyfenozide, a benzylhydrazide growth regulator (IGR) through leaf-dip bioassays^1^. Chlorfenapyr, spinosad, and indoxacarb were moderately toxic against 3rd instar larvae compared to the other tested insecticides.

Chlorantraniliprole, flubendiamide, and lufenuron also proved very toxic against 3rd instar *S. litura* larvae. Low to very low resistance was reported for Pakistani strains of the armyworm against chlorantraniliprole, flubendiamide, spinetoram, and spinosad^2^. Since the registration of lufenuron in Pakistan in 1996^30^, it gained popularity for successful control of the armyworm, however, moderate to high lufenuron resistance was reported in both *S. litura* and *S. exigua* in Pakistan^2,5^.

Before evaluating the efficacy of insecticide and EPN mixtures against *S. litura*, the toxic effects of the selected insecticides were first estimated against the two EPN spp., *S. carpocapsae,* and *H. indica* to appraise their compatibility with the tested insecticides. Our results indicate that all insecticides were relatively harmless or least toxic to the nematodes with mortalities less than 20% after 96 h of exposure. The insecticides caused less than 10% mortalities in both EPNs after 72 h of exposures, which encourages the integrated use of insecticides and EPNs as tank mixes are not usually prepared more than 24 h before spraying.

Studies have shown that delayed exposures of certain insecticides at higher concentrations can cause variable mortality in both EPN species (*S. carpocapsae* and *H. indica*)^12^, however, the insecticides remained harmless even after longer treatment exposition. Low EPN mortality was reported with certain other insecticides including chlorpyrifos^37^, whereas some conventional organophosphates including chlorphenvinphos and dichlorvos and their mixtures were reported to cause high mortality of *Heterorhabditis amazonensis*. Conversely, deltamethrin, and chlorpyrifos-ethyl did not reduce the survival of that EPN species^26,38^. *S. carpocapsae* was also reported to be more susceptible to deltamethrin compared to *H. bacteriophora* and *H. indica*^25^. Similarly, some commercial formulations of fipronil, malathion, cypermethrin, imidacloprid, chlorantraniliprol, and azadirachtin were also reported to cause no harm to survival or infectivity of entomopathogenic nematodes^35^.

In nematodes, the involvement of butyrylcholinesterase as a front line of defense was proposed because it may protect acetylcholinesterase from an early attack by its inhibitors^39^. Results of our study and some other reports suggest that several EPNs are compatible with many insecticides and hence can be used in combinations or mixtures in pest management programs^11–13,21,38^.

Synergistic, additive, and antagonistic interactions have been reported when some conventional and novel chemical insecticides were tested in combination with *S. carpocapsae* and *H. indica* after 48 h of treatment exposures. However, with increased exposure time to 96 h, the interactions of EPNs and insecticides turned antagonistic^25^. The increased exposure period to the EPNs alone and in combination with insecticides have proven to increase the mortality of insect larvae^11,12,38^. Similarly, an increase in rates of *H. indica* and *S. glaseri*, resulted in higher mortalities in *S. litura* under lab conditions, whereas *H. indica* demonstrated greater efficacy in greenhouse-pot-culture conditions^14^. Increased rates of *H. indica* and *S. carpocapsae* also produced greater mortality of *S. litura* larvae after 48 and 96 h of exposure^12,24^.

Combinations of *H. bacteriophora* with chlorantraniliprole and imidacloprid demonstrated synergistic or additive interactions in second-instar larvae of *Holotrichia oblita* and produced faster mortality than the EPNs or insecticide alone. Combination of *S. carpocapsae* with chlorantraniliprole or spinetoram has been suggested as a least toxic control strategy against the fall armyworm, *S. frugiperda*^15^.

It can be inferred from our results that all insecticides proved toxic alone and when applied in mixtures with EPNs against *S. litura* larvae. We suggest that more EPNs should be investigated for their compatibility with insecticides against certain insect pests of agricultural economic significance.

## Conclusions

Several reports describe the development of insecticide resistance in the armyworm *S. litura* against many insecticides included in pest management programs in many countries. Growers often apply huge volumes of insecticide to control resistant strains of pests without knowing their risks to the environment and to public health. Efforts are being made to investigate alternative methods to be used either alone or in combination with chemical insecticides for mitigation of this notorious pest. Entomopathogenic nematodes are among useful options for inclusion in IPM programs, used alone or in combination with chemical insecticides. The experiments conducted in the present study on compatibility of two EPN spp. with reduced doses of some novel reduced-risk chemical insecticides show significant potential for armyworm management. EPN mixtures with reduced volumes of insecticides will also help in lowering the risks of environmental pollution and to public health.

## Data Availability

Statistically nalysed data are presented in the manuscript. Observations recorded during the experiments are acquiesced to the Department of Entomology, University of Agriculture, Faisalabad, Pakistan and hence, the consent of authorities is required for obtaining the data.
